# State-Level Trends and Correlates for Cross-Sector Collaboration on School Nutrition and Physical Education Activities, 2000–2012

**DOI:** 10.5888/pcd13.160032

**Published:** 2016-07-21

**Authors:** Jennifer E. Pelletier, Melissa N. Laska, Richard MacLehose, Toben F. Nelson, Marilyn S. Nanney

**Affiliations:** Author Affiliations: Melissa N. Laska, Richard MacLehose, Toben F. Nelson, University of Minnesota, School of Public Health, Minneapolis, Minnesota; Marilyn S. Nanney, University of Minnesota, Department of Family Medicine and Community Health, Minneapolis, Minnesota.

## Abstract

**Introduction:**

Cross-sector collaboration on child obesity prevention is common, yet little research has examined the context of collaboration at the state level. This study describes secular trends in collaboration between state agency staff responsible for school nutrition and physical education activities and other organizations from 2000 to 2012.

**Methods:**

Data from the School Health Policies and Practices Study were used to describe collaboration between state agency staff and 13 types of public, private, and nonprofit organizations. Breadth of collaboration in 2012 was examined across political, social, and economic conditions.

**Results:**

Collaboration between state agency staff and other organization types increased from 2000 to 2006 and decreased or stabilized from 2006 to 2012. Breadth of collaboration was greater in states with a physical education coordinator, higher levels of poverty, higher prevalence of childhood obesity, and more public health funding. Breadth was similar across states by census region, political party of governor, majority party in state legislature, percentage non-Hispanic white population, high school graduation rate, and unemployment rate.

**Conclusion:**

Cross-sector collaboration on school nutrition and physical education was widespread and did not vary substantially across most political, social, and economic measures. Expanded monitoring and surveillance of state-level collaboration would assist efforts to understand how state agencies work across sectors and whether this collaboration affects the support they provide to schools.

## Introduction

The multifactorial nature of childhood obesity has led to calls for increased collaboration across multiple sectors of society to address the environmental, social, and policy factors driving the epidemic (1–4). Cross-sector collaborative partnerships, or groups of organizations from different sectors (eg, public agencies, community-based organizations, private businesses) have been particularly active in obesity prevention in primary and secondary school settings. These partnerships have successfully pursued shared goals such as developing comprehensive wellness policies and changing state policies (1,5,6).

Collaborative partnerships are theorized to be more effective in achieving broad social change than single-sector efforts (eg, public agencies) because they leverage the skills, knowledge, resources, and power of their member organizations (7,8). Cross-sector collaboration has been found to improve community capacity to make community-wide changes for obesity prevention by increasing community engagement and identifying opportunities for environmental and policy interventions (6,9–11). States and communities that engage cross-sector partners have also adopted more obesity-related policies (12,13) and more successfully implemented obesity-related policies and practices in schools and communities (5,13). Despite widespread emergence of collaborative partnerships for childhood obesity prevention during the past decade, little research has examined the conditions under which collaborative partnerships develop (14) and how cross-sector collaboration can be structured to achieve the greatest outcomes for obesity prevention (1,15,16).

Partnership breadth (the number of sectors or organization types participating in the partnership) is one structural feature that is theorized to allow partnerships to take on broad, comprehensive activities and increase the likelihood of effecting positive change (7,8,17). The primary aim of this study was to examine how organizational participation in state-level collaborative partnerships addressing school nutrition and physical education (PE) evolved from 2000 to 2012, an active period of policy and programmatic initiatives for obesity prevention in schools (18). A secondary aim of this study was to identify the political, social, and economic conditions associated with the breadth of state-level partnerships in 2012. We hypothesized that breadth would increase over time as collaborative partnerships matured and that breadth would vary across political, social, and economic characteristics of states (19).

## Methods

### Data

Collaboration was measured by using data from the nutrition services and physical activity questionnaires in the School Health Policies and Practices Study (SHPPS) (20), a survey administered to all 50 states and the District of Columbia by the Centers for Disease Control and Prevention (CDC) in 2000, 2006, and 2012. CDC staff instructed state contacts to identify the most knowledgeable state-level staff members (nutrition directors, commissioners, and consultants and PE directors, specialists, and consultants) on relevant topics to complete the questionnaires.

In the nutrition services questionnaire in 2000, a series of 8 questions asked whether state-level child nutrition or food service (CNFS) staff worked on school food service or nutrition activities during the previous 12 months with state-level school health education staff; school health services staff; school mental health or social services staff; PE staff; staff or members of a state-level health organization such as the American Heart Association or the American Cancer Society; a food commodity organization such as the Dairy Council or state produce growers association; businesses; or colleges or universities. In 2006 and 2012, five questions were added (questions on working with staff or members of the state department of agriculture; Action for Healthy Kids; a state-level school nurses association; a state-level physicians organization such as the American Academy of Pediatrics; or the state-level School Nutrition Association).

A similar series was asked in the physical activity questionnaire. In 2000, ten questions asked whether state-level PE staff worked on PE activities during the previous 12 months with state-level school health education staff; school health services staff; school mental health or social services staff; school nutrition or food service staff; or staff or members of the state parks or recreation department; the state-level American Alliance of Health, Physical Education, Recreation and Dance (AAHPERD); a state-level health organization such as the American Heart Association or the American Cancer Society; the Governor’s Council on Physical Fitness and Sports; businesses; or colleges or universities. In 2006 and 2012, three questions were added (questions on working with staff or members of Action for Healthy Kids; a state-level school nurses association; and a state-level physicians organization such as the American Academy of Pediatrics).

All questions were developed and reviewed by CDC staff, subjected to cognitive testing, and reviewed by external reviewers before their inclusion (20). Response options were yes, no, or no state-level staff in this area (for state-level staff questions only). Six states did not answer at least one question in the series in 2012, resulting in missing data for those questions. We coded responses as 1 (yes) or 0 (no/no state level staff/no answer); we assumed that lack of staff precluded collaboration and that no answer meant the respondent was unsure or unaware of collaboration. We repeated the analysis excluding the questions with missing data and had similar results.

Political, social, and economic characteristics found to predict state legislative activity on childhood obesity were examined as correlates of collaboration breadth (21,22). Because most state legislation on childhood obesity focuses on school settings (23), we hypothesized that contextual factors affecting legislative activity would also correlate with cross-sector collaboration on school nutrition and PE activities. Publicly available state-level measures were aligned with the period of SHPPS data collection (2011–2012). Measures (Table 1) included presence of a state-level coordinator for school nutrition or PE (SHPPS 2012); political affiliation of the governor and majority party in the state legislature (Council of State Governments, 2011–2012 session, compiled by the University of Kentucky Center for Poverty Research [UKCPR] [24]); unemployment rate (Bureau of Labor Statistics, 2011, compiled by UKCPR [24]; census region, percentage non-Hispanic white, poverty rate, and percentage of adults aged 25 or older with a high school education (2013 American Community Survey, 3-year estimates [25]); childhood obesity prevalence (2011–2012 National Survey of Children’s Health [26]); and total CDC funding and state public health budgets (Trust for America’s Health, fiscal year 2011 [27]). 

**Table 1:** Sources of Data for State Trends and Correlates for Cross-Sector Collaboration on School Nutrition and Physical Education Activities, 2000–2012

Data Source	Variable(s)	Description	Year(s)
**School Health Policies and Practices Study (20)**	Food service/nutrition collaboration	Yes/no questions assessed whether state-level nutrition or food service staff worked with public, private, or nonprofit entities on school food service or nutrition activities during previous 12 months	2000, 2006, 2012
	Physical activity/education collaboration	Yes/no questions assessed whether state-level physical education staff worked with public, private, or nonprofit entities on physical education activities during previous 12 months	
	Food service coordinator	Yes/no question asked whether someone in the state oversees or coordinates food service for schools, for example, a state food service director or director of child nutrition	
	Physical education coordinator	Yes/no question asked whether someone in the state oversees or coordinates physical education	

**University of Kentucky Center for Poverty Research (24)**	Political affiliation of governor	Dichotomized as Democrat or not a Democrat; data collected by Council of State Governments	2011–2012 session
	Majority party in state legislature	Democratic control of neither, one, or both houses; data collected by Council of State Governments	
	Unemployment rate	Annual average of percentage of labor force unemployed; computed by Bureau of Labor Statistics	2011

**US Census Bureau, American Community Survey (25)**	Census region	Northeast, Midwest, South, or West^a^	2013 (3-year average centered on 2012)
	Percentage non-Hispanic white	Percentage of individuals reporting non-Hispanic white race/ethnicity	
	Poverty rate	Percentage of individuals below federal poverty level	
	Rate of graduation from high school	Percentage of adults aged ≥25 y with a high school diploma or equivalent	

**National Survey of Children’s Health (26)**	Childhood obesity prevalence	Percentage of children aged 10–17 y with BMI ≥95th percentile, based on parent report in a nationally representative sample of households with children (one child randomly selected from each household)	2011–2012

**Trust for America’s Health (TFAH) (27)**	CDC funding	All CDC funding awarded to state and local health departments, universities, and public and private agencies; data provided to TFAH by CDC’s financial management office, in dollars	2011
	State public health budget	All state health funding (general revenue and dedicated funds, in dollars), except the following: Medicaid and the Children’s Health Insurance Program, comparable health insurance programs for low-income residents, mental health funds, services related to developmental disabilities or severely disabled persons, funds for the Special Supplemental Nutrition Program for Women, Infants, and Children (WIC), and state-sponsored pharmaceutical programs	

Abbreviation: BMI, body mass index; CDC, Centers for Disease Control and Prevention.

a The 4 census regions were defined according to the American Community Survey (25). Northeast: Connecticut, Maine, Massachusetts, New Hampshire, New Jersey, New York, Pennsylvania, Rhode Island, Vermont. Midwest: Illinois, Indiana, Iowa, Kansas, Michigan, Minnesota, Missouri, Ohio, Nebraska, North Dakota, South Dakota, Wisconsin. South: Alabama, Arkansas, Delaware, District of Columbia, Florida, Georgia, Kentucky, Louisiana, Maryland, Mississippi, North Carolina, Oklahoma, South Carolina, Tennessee, Texas, Virginia, West Virginia. West: Alaska, Arizona, California, Colorado, Hawaii, Idaho, Montana, Nevada, New Mexico, Oregon, Utah, Washington, Wyoming.

### Analysis

We counted the number of states collaborating with each organization type for 2000, 2006, and 2012 and calculated changes that occurred from 2000 to 2006 and from 2006 to 2012 using the comparable questions asked in both years. We defined collaboration breadth in each state as the sum of the number of organization types collaborating with 1) CNFS staff on school nutrition activities and 2) PE staff on PE activities. We examined average collaboration breadth in 2012 overall and stratified by state characteristics. To facilitate comparisons, we categorized continuous measures of state characteristics into tertiles. We reported means and 95% confidence intervals for collaboration breadth across strata of state characteristics. We did not conduct statistical tests for this descriptive analysis.

## Results

From 2000 to 2006, the number of states in which CNFS staff collaborated with other state-level school health staff increased (Table 2). In 2000, CNFS staff in 24 states reported collaborating with state-level PE staff, which increased to 40 states by 2006. Smaller increases were seen for collaboration between CNFS staff and state-level staff from health education (+8 states), mental health or social services (+7 states), and health services (+5 states). However, from 2006 to 2012, these increases were reversed (ie, fewer states reported collaborating) for all state-level school health staff except state-level PE staff, which decreased by only 3 states.

**Table 2:** Number of States Collaborating With Each Type of Organization on School Nutrition and Physical Education Activities, School Health Policies and Practices Study, 2000–2012

Type of Staff Member or Organization	No. of States in 2000	No. of States in 2006	No. of States in 2012	Change From 2000 to 2006	Change From 2006 to 2012
**Child nutrition or food service staff collaborated with . . .**					
State-level physical education staff	24	40	37	+16	−3
State-level school health education staff	40	48	43	+8	−5
State-level school health services staff	37	42	37	+5	−5
State-level school mental health or social services staff	19	26	19	+7	−7
Businesses	25	32	26	+7	−6
Academic institutions	46	48	45	+2	−3
State-level health organization such as the American Heart Association or the American Cancer Society	36	39	31	+3	−8
Action for Healthy Kids^a^	—b	48	41	—b	−7
State-level school nurses association	—b	35	36	—b	+1
State-level physicians organization such as the American Academy of Pediatrics	—b	29	16	—b	−13
State department of agriculture	—b	33	49	—b	+16
State-level school nutrition associations	—b	50	48	—b	−2
Food commodity organization, such as the Dairy Council or state produce growers association	49	48	49	−1	+1
**Physical education staff collaborated with . . . **					
State-level school nutrition or food service staff	21	45	40	+24	−5
State-level school health education staff	36	43	43	+7	0
State-level school health services staff	24	42	36	+18	−6
State-level school mental health or social services staff	17	25	22	+8	−3
Businesses	15	22	25	+7	+3
Academic institutions	36	45	44	+9	−1
State-level health organization, such as the American Heart Association or the American Cancer Society	31	39	39	+8	0
Action for Healthy Kids^a^	—b	42	35	—b	−7
State-level school nurses association	—b	32	34	—b	+2
State-level physicians organization, such as the American Academy of Pediatrics	—b	22	17	—b	−5
State parks or recreation department	12	21	22	+9	+1
Governor’s Council on Physical Fitness and Sports	21	30	21	+9	−9
State-level American Alliance of Health, Physical Education, Recreation and Dance	35	45	46	+10	+1

a Action for Healthy Kids is a national nonprofit organization that works with partner organizations to support physical activity and healthy eating in schools (www.actionforhealthykids.org).

b Question not asked in 2000 survey.

The number of states in which CNFS staff reported collaborating with staff or members of businesses and academic institutions increased from 2000 to 2006 and decreased from 2006 to 2012, leaving overall collaboration unchanged from 2000 to 2012. From 2006 to 2012, we found decreases in the number of states in which CNFS staff reported collaborating with nonprofit organizations, including state-level health organizations (−8 states), Action for Healthy Kids (−7 states), and state-level physicians organizations (−13 states). The only notable increase during this later period was the number of states in which CNFS staff reported collaborating with staff from the state department of agriculture (+16 states).

In 2006 and 2012, CNFS staff in most states reported collaborating with all organization types except school mental health or social services staff and staff or members of a state-level physicians organization. The most common collaborators throughout the study period were state-level health education staff (40–48 states), academic institutions (45–48 states), Action for Healthy Kids (41–48 states), state school nutrition associations (48–50 states), and food commodity organizations (48–49 states).

From 2000 to 2006, the number of states in which PE staff reported collaborating with each organization type increased by 7 to 24 states (Table 2). The greatest increases were for collaboration with state-level school nutrition or food service staff (+24 states) and state-level health services staff (+18 states). From 2006 to 2012, we found few increases and several notable decreases in the number of states in which PE staff reported collaborating with staff or members of nonprofit organizations, including the Governor’s Council on Physical Fitness and Sport (−9 states), Action for Healthy Kids (−7 states), and state-level physicians organizations (−5 states).

Compared with CNFS staff, PE staff were less likely to report collaborating with the following organization types in 2000: state-level staff from school health education, health services, and mental health or social services, and staff or members of businesses, academic institutions, or state-level health organizations. However, after the larger increases in collaboration reported by PE staff compared to CNFS staff from 2000 to 2012, collaboration with most organization types was similar for PE activities and school nutrition activities in 2012.

In 2012, the number of organization types working with CNFS staff on school nutrition activities (collaboration breadth) ranged from 4 to 13 of 13 organization types measured (Figure), with a median of 10. The number of organization types working with PE staff on PE activities ranged from 0 to 13, with a median of 9. PE staff from 3 states (Alaska, Rhode Island, and Wyoming) did not report collaborating with any organization types on PE activities.

**Figure:**
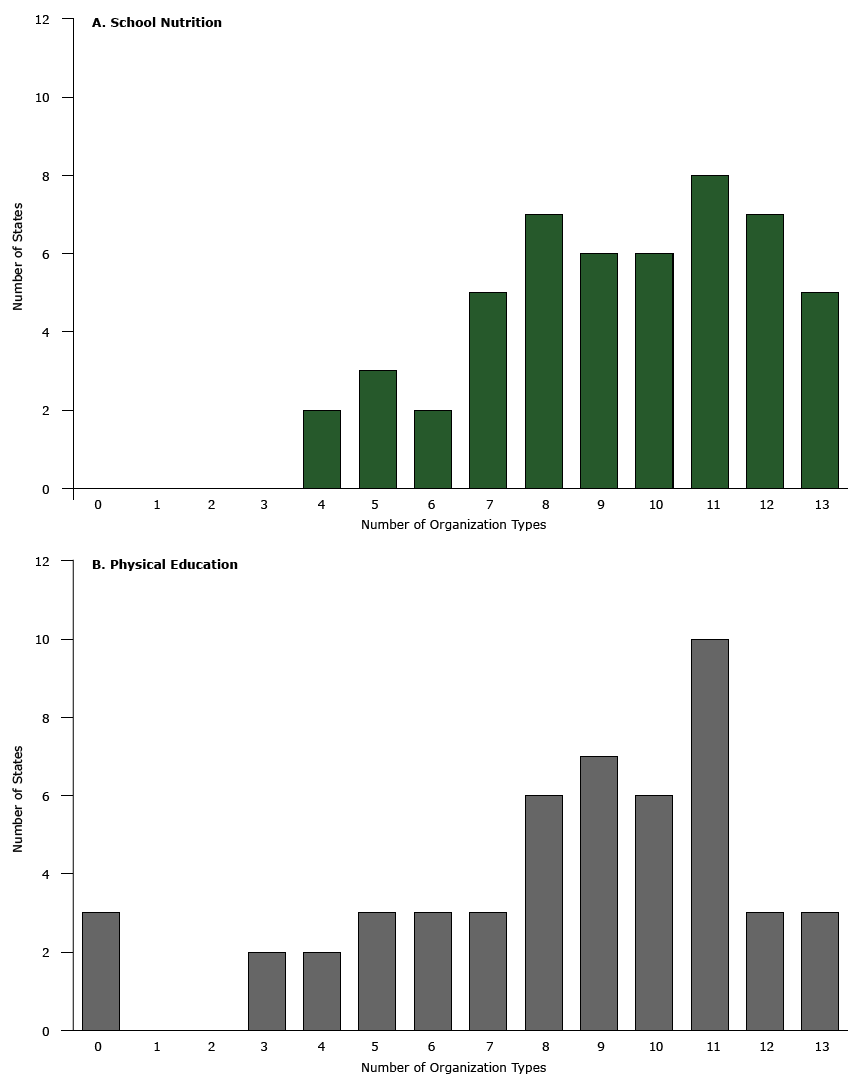
Distribution of number of organization types working with state agency staff on A) school nutrition and B) physical education activities, 50 states and District of Columbia, 2012. Source of data: School Health Policies and Practices Study (20). **Table:** 

No. of Organization Types	School Nutrition, No. of States	Physical Education, No. of States
0	0	3
1	0	0
2	0	0
3	0	2
4	2	2
5	3	3
6	2	3
7	5	3
8	7	6
9	6	7
10	6	6
11	8	10
12	7	3
13	5	3

Collaboration breadth for both school nutrition and PE activities did not vary substantially across most state characteristics (Table 3). In states with a state-level PE coordinator, we found an average collaboration breadth of 9.1 organization types, compared with 5.7 types in states without a PE coordinator. All but 2 states had a state-level school nutrition coordinator; this measure was excluded from analysis because there was not enough variation. For PE activities, we found higher collaboration breadth in states with the highest levels of childhood obesity (9.6 organization types) and poverty (9.8 organization types). Collaboration breadth for both school nutrition and PE was lowest among states with the lowest levels of CDC funding (8.4 and 6.9 organization types, respectively). States with the highest level of CDC funding had the greatest collaboration breadth for school nutrition (10.0 organization types). States with larger public health budgets also had higher collaboration breadth for PE (7.0 organization types in the lowest level, versus 8.9 in the middle level and 9.1 in the highest level).

**Table 3:** Selected State Characteristics and Number of Organization Types^a^ Engaged in Collaboration on School Nutrition and Physical Education Activities, United States, 2012

Characteristic	No. of States and District of Columbia	School Nutrition, Mean (95% CI), No. of Organization Types^a^	Physical Education, Mean (95% CI), No. of Organization Types^a^
**All states and District of Columbia**	51	9.4 (8.7–10.1)	8.3 (7.4–9.2)
**Census region^b^**			
Northeast	9	9.3 (7.5–11.1)	7.2 (4.5–9.9)
Midwest	12	10.1 (8.5–11.7)	8.1 (6.4–9.7)
South	17	9.4 (8.5–10.3)	9.6 (8.5–10.8)
West	13	8.6 (7.0–10.2)	7.5 (5.4–9.6)
**Government/Political**			
**Has a state-level physical education coordinator^c^ ^, d^**			
No	9	—e	5.7 (3.1–8.3)
Yes	41	—e	9.1 (8.3–9.9)
**Has a state-level nutrition coordinator^f^**			
No	2	—f	—e
Yes	49	—f	—e
**Majority party in state legislature^g^^, ^ h**			
Neither house has a Democratic majority	29	9.3 (8.3–10.2)	7.9 (6.6–9.3)
1 House has a Democratic majority	5	9.4 (6.9–11.9)	9.0 (6.8–11.2)
Both houses have a Democratic majority	15	9.5 (8.3–10.8)	8.6 (7.0–10.2)
**Political affiliation of governor^g^^, ^ h**			
Not Democrat	30	9.2 (8.3–10.0)	8.3 (6.9–9.6)
Democrat	20	9.8 (8.6–10.9)	8.4 (7.2–9.5)
**Sociodemographics**			
**Percentage of population that is non-Hispanic white, by tertile^i^**			
Lowest	18	9.2 (8.0–10.3)	8.3 (6.9–9.7)
Middle	16	10.1 (8.9–11.2)	9.1 (7.5–10.6)
Highest	17	8.9 (7.6–10.2)	7.6 (5.8–9.5)
**Percentage of adults who have a high school education, by tertile^i^**			
Lowest	17	9.4 (8.2–10.5)	9.0 (7.4–10.6)
Middle	17	9.8 (8.5–11.1)	8.4 (7.2–9.5)
Highest	17	8.9 (7.7–10.1)	7.6 (5.7–9.5)
**Prevalence of childhood obesity, by tertile^j^**			
Lowest	17	8.9 (7.6–10.3)	7.9 (6.1–9.7)
Middle	17	9.9 (8.8–11.0)	7.4 (5.9–9.0)
Highest	17	9.2 (8.1–10.4)	9.6 (8.4–10.9)
**Economic**			
**Poverty rate, by tertile^i^**			
Lowest	17	9.4 (8.1–10.7)	7.9 (6.1–9.6)
Middle	17	8.9 (7.6–10.2)	7.2 (5.6–8.9)
Highest	17	9.8 (8.8–10.7)	9.8 (8.7–10.9)
**Unemployment rate, by tertile^k^**			
Lowest	17	9.4 (8.2–10.6)	7.6 (5.9–9.2)
Middle	17	9.2 (7.9–10.4)	8.7 (7.1–10.3)
Highest	17	9.5 (8.3–10.6)	8.6 (7.1–10.2)
**Funding from Centers for Disease Control and Prevention, by tertile^k^**			
Lowest	17	8.4 (7.1–9.6)	6.9 (4.8–9.0)
Middle	17	9.7 (8.7–10.7)	10.2 (9.4–10.9)
Highest	17	10.0 (8.6–11.4)	7.9 (6.6–9.2)
**State public health budget, by tertile^k^**			
Lowest	17	9.1 (7.9–10.3)	7.0 (4.9–9.1)
Middle	17	9.6 (8.3–10.9)	8.9 (7.7–10.0)
Highest	17	9.4 (8.1–10.6)	9.1 (7.8–10.3)

Abbreviation: CI, confidence interval.

a The School Health Policies and Practices Study (SHPPS) (20) was used to describe collaboration between state agency staff and 13 types of public, private, and nonprofit organizations. For nutrition activities, the 13 organization types were as follows: 1) state-level school health education staff; 2) school health services staff; 3) school mental health or social services staff; 4) physical education staff or nutrition staff; 5) staff or members of a state-level health organization such as the American Heart Association or the American Cancer Society; 6) a food commodity organization such as the Dairy Council or state produce growers association; 7) businesses; 8) colleges or universities; 9) state department of agriculture; 10) Action for Healthy Kids; 11) state-level school nurses association; 12) state-level physicians organization such as the American Academy of Pediatrics; and 13) state-level School Nutrition Association. For physical education activities, the 13 types were as follows: 1) state-level school health education staff; 2) school health services staff; 3) school mental health or social services staff; 4) school nutrition or food service staff; 5) staff or members of the state parks or recreation department; 6) the state-level American Alliance of Health, Physical Education, Recreation and Dance; 7) a state-level health organization such as the American Heart Association or the American Cancer Society; 8) the Governor’s Council on Physical Fitness and Sports; 9) businesses; 10) colleges or universities; 11) Action for Healthy Kids; 12) state-level school nurses association; and 13) state-level physicians organization such as the American Academy of Pediatrics.

b The 4 census regions were defined according to the American Community Survey (25). Northeast: Connecticut, Maine, Massachusetts, New Hampshire, New Jersey, New York, Pennsylvania, Rhode Island, Vermont. Midwest: Illinois, Indiana, Iowa, Kansas, Michigan, Minnesota, Missouri, Ohio, Nebraska, North Dakota, South Dakota, Wisconsin. South: Alabama, Arkansas, Delaware, District of Columbia, Florida, Georgia, Kentucky, Louisiana, Maryland, Mississippi, North Carolina, Oklahoma, South Carolina, Tennessee, Texas, Virginia, West Virginia. West: Alaska, Arizona, California, Colorado, Hawaii, Idaho, Montana, Nevada, New Mexico, Oregon, Utah, Washington, Wyoming.

c Data were missing for Rhode Island.

d Source of data: School Health Policies and Practices Study (SHPPS) (20).

e Does not apply.

f Data on state nutrition coordinator were omitted from analysis because 49 of 51 states reported having a state nutrition coordinator.

g Source of data: University of Kentucky Center for Poverty Research (24).

h Nebraska was excluded from the analysis on affiliation of state legislature because it has a unicameral, nonpartisan legislature, and the District of Columbia was excluded from the analyses on affiliation of state legislature and governor affiliation because it is governed by a city council and mayor.

i Source of data: American Community Survey (25).

j Source of data: National Survey of Children’s Health (26).

k Source of data: Trust for America’s Health (27).

## Discussion

Our study is the first to quantify the extent to which various organization types are involved in state-level school nutrition and PE activities across the country. Collaboration between CNFS and PE staff and other organization types increased from 2000 to 2006, and decreased or stabilized from 2006 to 2012 for all organization types except state departments of agriculture. This trend is consistent with theories and research on collaborative partnerships, which posit that the breadth of collaborative networks expands as they mature (7,28) but may reach a threshold beyond which additional partners add little benefit and may hinder agreement on goals (17). This trend may also have resulted from external economic and political factors. The increases from 2000 to 2006 may reflect states’ efforts to support school districts’ development of federally mandated wellness policies from 2004 to 2006 (29), whereas the increase in collaboration with state departments of agriculture from 2006 to 2012 may reflect greater regulatory flexibility and funding for farm-to-school programming provided by the 2008 Farm Bill (30) and the 2010 Healthy Hunger-Free Kids Act (31).

The economic recession that began in 2008 had substantial impacts on state budgets, possibly reducing state agencies’ capacity to engage large numbers of stakeholders. On the other hand, federal stimulus funding through the American Recovery and Reinvestment Act of 2009 (32) and CDC’s Community Transformation Grants (33) from 2011 through 2014 provided new sources of funding to state health departments for prevention activities. Indeed, the relationship between funding and collaboration breadth is unclear. Our study found that states receiving the lowest levels of CDC funding collaborated with fewer organization types on school nutrition and PE activities in 2012. One study found no association between several measures of state and federal funding for population health and policy enactment from 2003 to 2005 (21). Another study found that states receiving CDC funding designated for building partnerships and capacity for obesity prevention activities enacted twice as many obesity-related laws in 2005 as states that did not receive such funding (34). Further research is needed to understand whether overall public health funding levels or funding targeted to obesity prevention is more effective in enabling states to engage a broad group of organizational stakeholders on obesity prevention activities.

Our study also sheds light on the most common organization types engaged in state-level school nutrition and PE activities during the past decade. These organizations include school nutrition and PE professional associations and food commodity organizations, which have a self-interest in school policies and practices, and academic institutions, which may partner on research and evaluation. In contrast, physicians associations and mental health and social service staff were among the least frequently reported collaborators; states may be missing opportunities to integrate school nutrition and PE activities with the activities of experts in such areas as weight stigma and eating disorders. Knowing which organizations are involved in obesity-related collaborative activities is important when designing and interpreting studies of collaboration impact because organizations may have different or even contradictory interests in the outcomes of collaborative activities. For example, public health advocacy organizations may have an interest in developing stronger state policies on obesity prevention, whereas organizations representing the food and beverage industry may have an interest in limiting the impact of these policies on sales of their products. 

Of particular interest to public health practitioners is the expansion across the country in collaboration between state-level school health staff from diverse disciplines, especially the large increases in the number of states in which state-level CNFS and PE staff work with each other on both topics. State agency staff are often responsible for coordinating implementation activities, including providing resources, training, and technical assistance to schools and districts (35), and greater collaboration among these staff may indicate adoption of a more integrated approach to obesity prevention and wellness in schools. Whether increased collaboration across departments has resulted in more effective or useful supports for implementation of federal and state policies at the school level is an important question for future research.

Several patterns in collaboration across state characteristics warrant discussion. States with a state-level PE coordinator engaged in collaboration with an average of 3.4 more organization types than states without a coordinator. Although we cannot determine from the SHPPS data the role of state-level PE coordinators in developing and managing cross-sector collaboration, theory and practice suggest that having an individual or organization act as a convener or coordinator is an important component of collaboration formation and effectiveness, and these are natural roles for state PE coordinators (36). Interestingly, nearly all states had a state-level school nutrition coordinator, which perhaps reflects the greater historical emphasis on school nutrition and food service policies than on PE and physical activity. A recent analysis found that state agencies provided more types of implementation support to schools and school districts for nutrition and food service than for PE and physical activity in 2012 (K. Grannon, MPH, et al., unpublished data, May 2016), which may reflect PE laws that are weak and nonspecific in most states (37). To create school environments that support healthy weight, states should pursue a comprehensive approach to changing school policies and practices addressing both nutrition and physical activity.

States with higher levels of poverty and childhood obesity also had broader collaboration on PE activities, which could indicate greater mobilization of diverse sectors for childhood obesity prevention in states where the rate of childhood obesity is highest. A complex health problem is a strong motivator for cross-sector collaboration; however, poverty, lack of funding, and other social issues may limit the effectiveness of collaboration (16).

This study illustrates both the strengths and limitations of using surveillance data to conduct research studies. Few data sources exist that enable examination of how partnerships change over time. The use of surveillance data in this study enabled a nationally representative descriptive analysis of organizations that worked together in each state in the country, providing insight into how our society and government are responding to the societal challenge of childhood obesity. Examining these questions is an important first step in identifying potential missed opportunities for partnership and generating hypotheses on the impact of these partnerships. The collaboration variables generated by the SHPPS may also be used as predictors or covariates in future studies investigating the relationships among collaboration, state policies, state agency support, and student health outcomes.

However, surveillance data are generally not collected with the same frequency or intensity as data collected for a specific research question and may be subject to measurement error resulting from respondents’ incomplete or inaccurate reports. The SHPPS data did not include measures of the strength or quality of relationships between organization types, and the survey had a limited list of potential collaborating organizations. SHPPS should consider adding questions on the frequency of interactions and purpose of collaboration (eg, implementation, evaluation) to assist in evaluating the impact of collaboration.

Cross-sector collaboration on school nutrition and PE was widespread and did not vary substantially across most political, social, and economic measures. Expanded monitoring and surveillance of state-level collaboration would assist in understanding how state agencies and departments work across sectors on obesity prevention activities and the impact collaboration may have on the types of support they provide to schools.
